# Antibacterial efficacy of peracetic acid in comparison with sodium hypochlorite or chlorhexidine against *Enterococcus faecalis* and *Parvimonas micra*

**DOI:** 10.1186/s12903-022-02148-8

**Published:** 2022-04-09

**Authors:** Benjamín Briseño-Marroquín, Angelika Callaway, Natascha Gol Shalamzari, Thomas Gerhard Wolf

**Affiliations:** 1 Department of Restorative, Preventive and Pediatric Dentistry, School of Dental Medicine, University of Bern, Freiburgstrasse 7, 3010 Bern, Switzerland; 2 Department of Periodontology and Operative Dentistry, University Medical Center of the Johannes Gutenberg-University, Mainz, Germany

**Keywords:** Bactericidal effect, *E. faecalis*, *P. micra*, Peracetic acid

## Abstract

**Background:**

The main goal of an endodontic treatment is a complete debridement of the root canal system; however, currently mechanical shaping and chemical cleaning procedures for this purpose have deemed non-satisfactory.

**Methods:**

The efficacy of peracetic acid (PAA; 0.5, 1.0, 2.0%), as a root canal irrigation solution, against *Enterococcus faecalis* (DSM 20478) and *Parvimonas micra* (DSM 20468) when compared with the one of sodium hypochlorite (NaOCI; 1.0, 3.0, 5.0%), chlorhexidine digluconate (CHX; 0.12, 0.2, 2.0%) and 0.9% NaCI (as a control solution) was in vitro investigated with the agar diffusion and direct contact methods. The inhibition zone diameters observed with the agar diffusion test were determined. The viable bacterial counts (CFU/ml) were calculated with the direct method.

**Results:**

The agar diffusion test showed that all three root canal irrigation solutions had an efficacy against *E. faecalis* at all concentrations. The largest inhibition zone diameters against *E. faecalis* were observed with 5.0% NaOCI. At all three concentrations of PAA, NaOCI, and CHX, the inhibition zone diameter increased with increase in concentration. For *P. micra*, PAA had a similar inhibition zone diameter despite a concentration increase. In contrast, for NaOCI and CHX, the inhibition zone diameter increased with increasing concentration. 2.0% CHX produced the largest inhibition zone diameter against *P. micra*. For *E. faecalis*, only the comparison between 2.0% PAA and 5.0% NaOCI showed statistical significance (*p* = 0.004). For *P. micra* the efficacy comparison between the lowest, middle, and highest concentrations of each solution, a statistical significance (*p* < 0.05) was found for all three solutions. After direct contact with PAA, NaOCI and CHX, no viable bacteria could be determined for either *P. micra* or *E. faecalis*.

**Conclusions:**

PAA had a similar antibacterial efficacy as the one of NaOCl and CHX when in direct contact with *E. faecalis* and *P. micra*. In the agar diffusion test, PAA showed a similar antibacterial efficacy as the one of CHX and a lower one as the one of NaOCl for *E. faecalis*.

## Background

Pulp tissue decomposition, thus, a bacterial colonization commonly derived from pulp inflammation develops into a complex microbial flora, either as non- or adherent aggregates to the dentinal walls [1]. Thus, two main goals of endodontic treatment are a complete pulp tissue and/or bacteria removal and to create an hermetical seal of the root canal system; hence, to produce ideal conditions for further inflammation prevention and/or healing of the periapical tissues. Therefore, a disinfecting root canal irrigation solution, as an enhancement of the mechanical procedures, is essential in order to achieve an effective bacterial removal from the root canal system. However, actual mechanical and chemical preparation procedures have deemed as insufficient [2]. Root canal irrigants ideally should have a broad antimicrobial spectrum, be efficient against obligately anaerobic and facultatively anaerobic microorganisms/biofilms, inactivate endotoxins, dissolve pulp tissue, avoid smear layer formation during instrumentation and should not irritate the periodontal tissues [3]. For that reason, actual root canal irrigating solutions are able to disinfect and to dissolve tissue (NaOCI; 2.5–6%), to enhance smear-layer removal EDTA (17%) and, if required, to act as bactericidal substantivity (CHX; 2.0%) [2]. It should constantly be also taken into account that biofilms are less sensitive to antimicrobial agents [4]. Therefore, a sufficient concentration and exposure time of the irrigating solution must be provided to dissolve such biofilms [5]. Furthermore, it has been reported [6] that CHX is not able to penetrate into deep layers of thick biofilms, thus, having a higher bactericidal effect on fresh biofilms than on mature ones. The antimicrobial potential of peracetic acid (PAA) as a disinfectant during root canal irrigation has been reported in earlier studies [7–10]. However, it has also been reported [10] that 10% PAA was not able to dissolve organic tissue; yet, it was also reported that 2.25% PAA combined with 1% NaOCl were able to demineralize root canal dentin [11].

Endodontic microflora is diverse and can consist of over 460 taxonomic groups [12]. *Enterococcus faecalis* and *Parvimonas micra* are two endodontic pathogens which have been isolated from persistent apical lesions, secondary infections, and periodontal lesions [13]. *E. faecalis* has a dentin penetration ability, is able to survive for relatively long periods of time [14], it can be consistently found in secondary and occasionally in primary endodontic infections [13, 15] and it can form biofilms in the root canal system [16]. *E. faecalis* can be eliminated from the root canal through irrigating solutions/gels such as NaOCI and CHX of different concentrations [7, 17] and with 1.0% PAA alone or in combination with 2.5% NaOCI [7]. *Parvimonas micra* is a Gram-positive, obligately anaerobic and small bacterium (0.3–0.7 µm) which is often present in pairs or in chains [18]. It has been found in pulp chambers of intact teeth with necrotic pulps [19], in primary endodontic infections with mixed flora [20], in the apical root region of extracted teeth [21] and root canals [22]. Thus *P. micra* can periodontally invade the root canal system causing primary endodontic infections, and is major pathogen obtained from inflamed pulp tissue samples [23]. Hence, the aim of this study was to investigate the antibacterial efficacy of PAA against *Enterococcus faecalis* and *Parvimonas micra* with an in vitro research model. Its bactericidal potential was compared with one of the two routinely employed root canal irrigation solutions: NaOCl and CHX at different concentrations.

The study hypothesis established that PAA would have a similar bactericidal potential against *E. faecalis* and *P. micra* as the one of NaOCl or CHX.

## Methods

### Materials and bacterial strains

Peracetic acid (PAA), sodium hypochlorite (NaOCl), and chlorhexidine digluconate (CHX) were chosen as test solutions in this in vitro study and sterile saline served as control solution. Concentrations of 0.5, 1.0 and 2.0% were prepared from 38 to 20% PAA (Merck KGaA, Darmstadt, Germany), concentrations of 1.0, 3.0 and 5.0% were prepared from 12% NaOCl (Carl Roth GmbH + Co. KG, Karlsruhe, Germany) and concentrations of 0.2 and 2.0%, were obtained from 20% CHX (Sigma Chemicals, St. Louis, MO, USA). A commercial oral rinse (Paroex, Sunstar, Kriftel, Germany) was used for a 0.12% CHX solution research group. For each independent experiment (n = 6) and each concentration, 5 ml stock solutions were freshly prepared for the three experimental solutions, using sterile distilled water. They were subsequently sterilized by passing through a filter (0.2 µm pore size, Minisart, Sartorius, Göttingen, Germany), and then stored in a refrigerator (12 °C). The potential bactericidal effects of the three solutions were tested using two bacterial strains belonging to species commonly found in endodontic infections. The facultatively anaerobic *Enterococcus faecalis* DSM 20478 and the obligately anaerobic *Parvimonas micra* DSM 20468, both the type strains of the species, were obtained lyophilized from the Leibniz Institute German Collection of Microorganisms and Cell Cultures (DSMZ, Braunschweig, Germany).

### Bacterial cultures

*E. faecalis* 20478 was grown as liquid culture in Schaedler bouillon (BBL Schaedler Broth, Becton Dickinson and Company, Sparks, MD, USA) or on Schaedler agar plates. *P. micra* 20468 was grown in Anaerobe Basal Broth (Oxoid Ltd., Basingstoke, Hampshire, UK) and on agar plates prepared from the broth. Anaerobic conditions were obtained through incubation of the bacterial cultures in an anaerobic jar containing a GasPak envelope producing H_2_ and CO_2_ (GasPak EZ; Becton Dickinson and Company, Sparks, MD, USA) for 24 h (*E. faecalis* 20478) or 72 h (*P. micra* 20468), at 37 °C (Heratherm Incubator, Thermo Scientific, Langenselbold, Germany). An inoculum of 400 µl was used for the liquid cultures. Purity of the bacterial cultures was confirmed each time by taking a sample, placing it on a microscope slide, and visually analyzing it using a phase contrast microscope (Carl Zeiss, Jena, Germany; 1250x). A modified agar diffusion test, the inhibition zone was used to obtain preliminary information about a potential bactericidal effect of the three solutions as already described [24]. 100 µl of the bacterial cultures were applied to the respective agar plates with a spreader, using a turntable. Then sterile paper discs (Oxoid Ltd, Basingstoke, Hampshire, UK) were placed on top of the agar, and 10 µl each of 0.5, 1.0 and 2.0% PAA, 1.0, 3.0 and 5.0% NaOCl and 0.12, 0.2 and 2.0% CHX were applied to the paper discs. After incubation for 24 or 72 h, depending on the bacterial strain as described above, the diameters of the inhibition zones were measured and expressed in millimeters.

In order to bring the bacterial cells into close contact with the antibacterial agent, aliquots of 1 ml for each agent, dilution, and bacterium were taken from liquid cultures, placed into sterile Eppendorf tubes (Eppendorf, Wesseling-Berzdorf, Germany) and centrifuged (Zentrifuge 5410, Eppendorf, Wesseling-Berzdorf, Germany). The supernatants were discarded and the pellets were washed twice with sterile saline to remove components from the growth media. Then 1 ml of test solution or saline a control was added, and mixed on a vortex (Janke & Kunkel IKA-Labortechnik, Staufen, Germany) to obtain a suspension. After 5 min of contact with PAA, CHX or saline as control, the suspensions were centrifuged again, following which the supernatants discarded, and then the pellets were washed once with sterile saline. 1 ml saline was added again and mixed as described above. In the case of exposure to NaOCl, no pellet was formed, thus, the washing step had to be omitted. Next, serial dilutions of 10^–1^ dilution steps were performed until a dilution factor of 10^–6^ was reached. 100 µl of each dilution were then plated on the respective agars and incubated as described above. The number of colonies on plates containing 30–300 colonies was then counted and converted to the number of organisms per milliliter (CFU). The number of viable bacteria (CFUs) for the untreated controls was considered as 100% survival or 0% reduction rate. The number of CFUs of the treated bacteria were then supposed to be compared with the controls, and reduction rates in percent to be calculated for each strain and each exposure.

### Statistical evaluation

A descriptive statistical evaluation for the numerical continuous variable size of the inhibition zones (mean, median, standard deviation, minima, maxima and percentile) was carried out (SPSS Version 22 for Windows; Chicago, IL, USA; Institute for Medical Biometry, Epidemiology and Informatics, IMBEI, Mainz, Germany). To compare the effects of the three different dilutions per solution, the nonparametric Friedman test for more than two dependent samples was used. The effects of the three different solutions (PAA, NaOCl or CHX) were compared for each bacterial strain by means of the nonparametric test for independent samples using the Kruskal–Wallis test (*p* < 0.05). The means, standard deviations, and medians were also determined for the colony forming units (Excel 2013; Microsoft, Seattle PO, USA). For comparison of the number of viable bacteria (untreated controls) and those after treatment with 0.5, 1.0 and 2.0% PAA, 1.0, 3.0 and 5.0% NaOCl or 0.12, 0.2 and 2.0% CHX the nonparametric Wilcoxon test for related samples was used (*p* < 0.05).

## Results

**Fig. 1:**
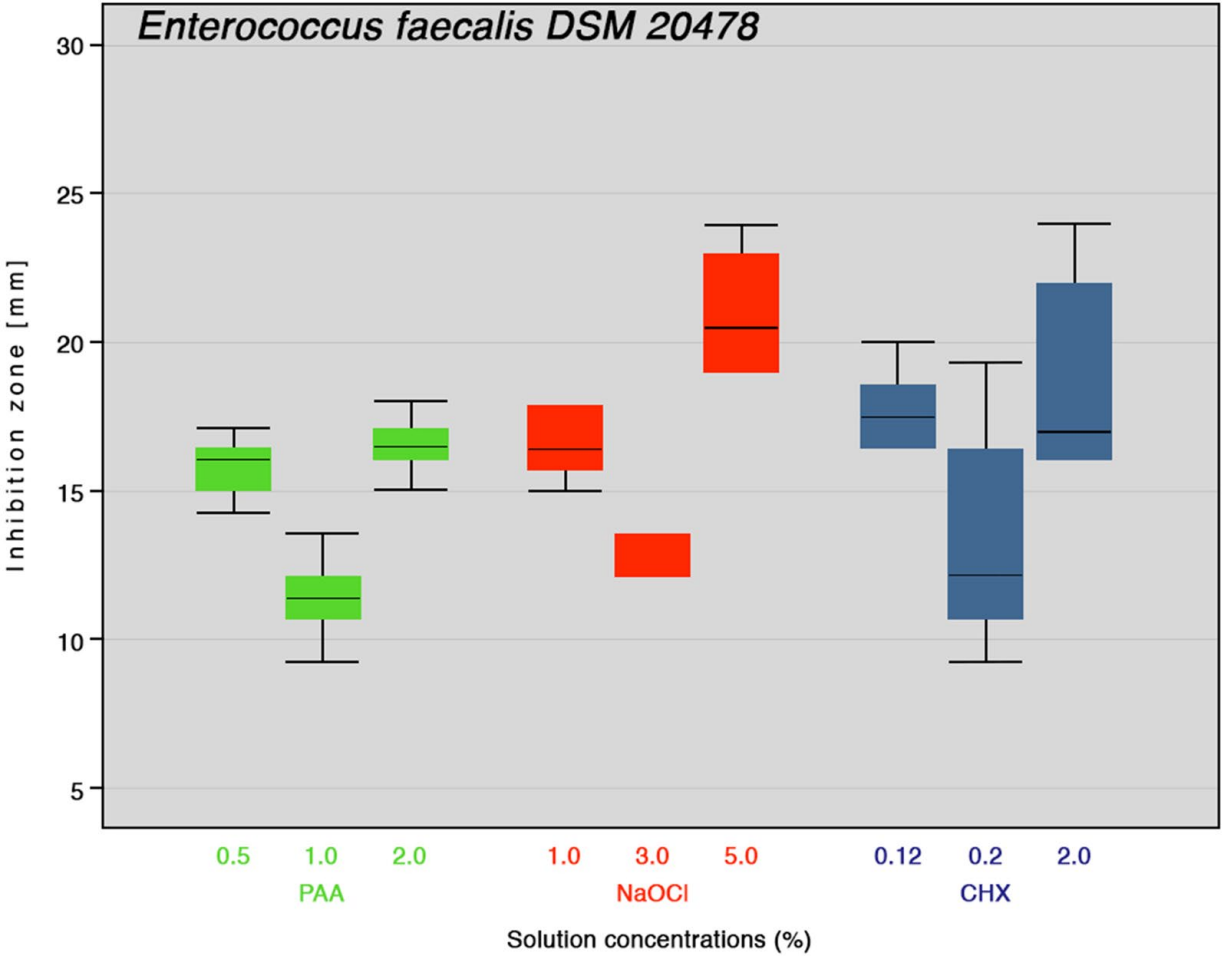
Inhibition zone diameters boxplot of different concentrations of peracetic acid (PAA), sodium hypochlorite (NaOCl) and chlorhexidine digluconate (CHX) against *E. faecalis* (n = 6). Medians are shown as lines inside the boxes, 25th and 75th percentiles as boxes, maximum and minimum values as whiskers, and outliers as circle on the plot

**Fig. 2:**
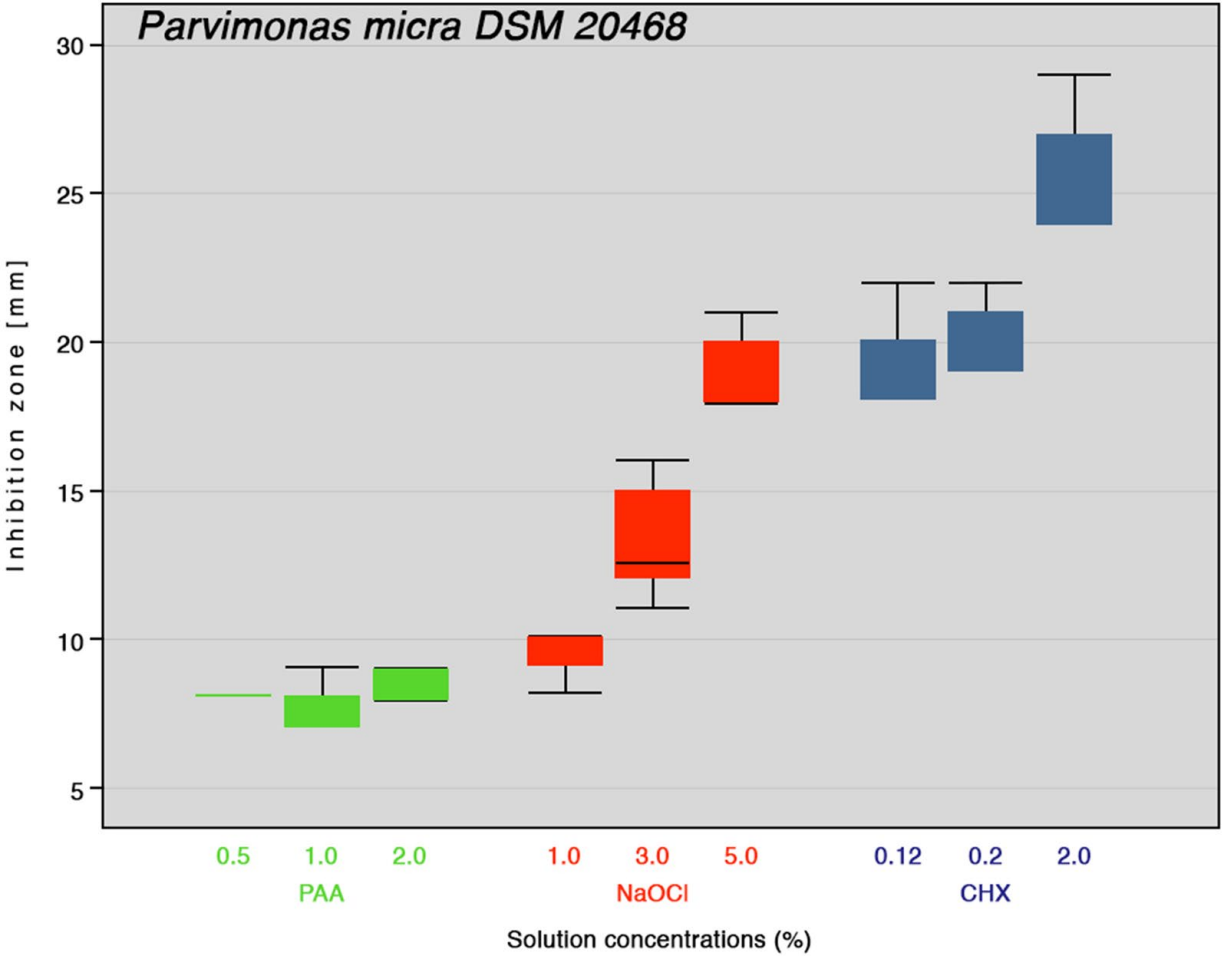
Inhibition zone diameters boxplot of different concentrations of peracetic acid (PAA), sodium hypochlorite (NaOCl) and chlorhexidine digluconate (CHX) against *P. micra* (n = 6). Medians are shown as lines inside the boxes, 25th and 75th percentiles as boxes, maximum and minimum values as whiskers, and outliers as circle on the plot

After 24 or 72 h of incubation inhibition zones, formed by peracetic acid (PAA), sodium hypochlorite (NaOCl) and chlorhexidine digluconate (CHX) at different concentrations against *E. faecalis* and *P. micra* were determined. No inhibition zones were formed in the negative control with 0.9% NaCI. A correlation was found between the increase in concentration and the inhibition zone diameter. 5.0% NaOCl and 2.0% CHX had similar antibacterial activity against *E. faecalis* (Fig. 1). The bactericidal effect of 2.0% PAA was similar to the respective CHX and NaOCl concentrations. However, all PAA concentrations exhibited the smallest inhibition zones against *P. micra* (Fig. 2). The results of the descriptive statistical evaluation of the inhibition zones and significant differences between the investigated concentrations of PAA, NaOCl and CHX against *E. faecalis* and *P. micra* are shown in Table 1. For both bacterial strains investigated, no colony forming units (CFUs) could be determined after direct contact with all three concentrations of PAA (0.5, 1.0 and 2.0%), NaOCl (1.0, 3.0 and 5.0%) and CHX (0.12, 0.2 and 2%); the *E. faecalis* controls ranged from 0.84 to 2.55 × 10^8^ CFU/ml and the *P. micra* controls ranged from 1.10 to 2.77 × 10^8^ CFU/ml (Table 2).

**Table 1:** Mean values, standard deviations (SD; ±), medians (M), minima (Min), and maxima (Max) of the inhibition zones (mm; n = 6) for the investigated strains: *E. faecalis* 20478, and *P. micra* 20468 produced by different concentrations of the solutions of peracetic acid (PAA), sodium hypochlorite (NaOCl) and chlorhexidine digluconate (CHX)

	Control	*Enterococcus faecalis* DSM 20478		
		PAA 0.5, 1, 2%	NaOCl 1, 3, 5%	CHX 0.12, 0.2, 2%
Mean	0	9.17, 12.5*, 16.5*	10.17, 14.17*, 21.0*	11.83, 13.83, 18.67
SD ( ±)	0	1.47, 1.04, 1.04	1.8, 1.47, 2.09	1.94, 2.63, 3.5
M	0	9.5, 12.5, 16.5	10.0, 14.0, 20.5	11.5, 13.0, 17.0
Min	0	7.0, 11.0, 15.0	8.0, 13.0, 19.0	10.0, 11.0, 16.0
Max	0	11.0, 14.0, 18.0	12.0, 17.0, 24.0	15.0, 18.0, 24.0

	Control	*Parvimonas micra* DSM 20468		
		PAA 0.5, 1, 2%	NaOCl 1, 3, 5%	CHX 0.12, 0.2, 2%
Mean	0	7.83*, 7.83*, 8.83*	9.5*, 13.17*, 19.17*	19.67*, 19.17*, 25.83*
SD ( ±)	0	0.4, 0.75, 0.51	1.3, 1.94, 1.16	1.5, 2.4, 1.94
M	0	8.0, 8.0, 8.0	9.0, 12.5, 19.0	20.0, 19.0, 25.0
Min	0	7.0, 7.0, 8.0	8.0, 11.0, 18.0	18.0, 15.0, 24.0
Max	0	8.0, 9.0, 9.0	12.0, 16.0, 21.0	22.0, 22.0, 29.0

Statistically significant differences when considering the inhibition zone size were observed for all three concentrations of the three solutions for *E. faecalis*: PAA (*p* = 0.002), NaOCl (*p* = 0.002) and CHX (*p* = 0.006) and for *P. micra*: NaOCl (*p* = 0.002), CHX (*p* = 0.011); yet not for PAA. When individually considering the lowest (0.5% PAA, 1.0% NaOCl and 0.12% CHX), medium (1.0% PAA, 3.0% NaOCl and 0.2% CHX) and highest (2.0% PAA, 5.0%, NaOCl and 2.0% CHX) concentrations of the three solutions for the two bacteria, statistically significant differences were obtained for *E. faecalis*: only between 1.0% PAA vs. 3.0% NaOCl, and 2.0% PAA vs. 5.0% NaOCl and for *P. micra*: between all three concentrations and irrigating solutions (*p < 0.05)

**Table 2:** CFUs of the control (untreated culture), after being in contact with the respective solutions of peracetic acid (PAA; 0.5, 1.0 and 2.0%), sodium hypochlorite (NaOCl; 1.0, 3.0 and 5.0%) and chlorhexidine digluconate (CHX; 0.12, 0.2 and 2%) and reduction rates in percent achieved by the respective antibacterial solutions

	Control	*Enterococcus faecalis* DSM 20478					
		PAA	PAA (%)	NaOC1	NaOCl (%)	CHX	CHX (%)
		0.5, 1, 2%		1, 3, 5%		0.12, 0.2, 2%	
Mean	15.6 × 10^7^	0.0	100.00	0.0	100.00	0.0	100.00
SD ( ±)	5.16	0.0	100.00	0.0	100.00	0.0	100.00
M	14.7 × 10^7^	0.0	100.00	0.0	100.00	0.0	100.00
Min	8.4 × 10^7^	0.0	100.00	0.0	100.00	0.0	100.00
Max	25.5 × 10^7^	0.0	100.00	0.0	100.00	0.0	100.00

	Control	*Parvimonas micra* DSM 20468					
		PAA	PAA (%)	NaOC1	NaOCl (%)	CHX	CHX (%)
		0.5, 1, 2%		1, 3, 5%		0.12, 0.2, 2%	
Mean	16.9 × 10^7^	0.0	100.00	0.0	100.00	0.0	100.00
SD ( ±)	5.09	0.0	100.00	0.0	100.00	0.0	100.00
M	17.8 × 10^7^	0.0	100.00	0.0	100.00	0.0	100.00
Min	11.0 × 10^7^	0.0	100.00	0.0	100.00	0.0	100.00
Max	27.7 × 10^7^	0.0	100.00	0.0	100.00	0.0	100.00

The calculated bactericidal rate for *E. faecalis* and *P. micra* with the three solutions at all concentrations was 100%; no CFUs were observed in any trial (SD = standard deviation; M = median; Max = maxima; Min = minima; n = 6)

## Discussion

A common disadvantage of in vivo and ex vivo research methods is that in a clinical situation a large number of different bacteria could be present, thus, the results obtained can only be transferred to a specific clinical condition to a limited extent. Ex vivo research models, either human or bovine teeth [6, 7, 9, 14, 25] are usually inoculated after sterilization and having attempted to standardize the investigation parameters. The advantage of these methodologies is that they resemble an in vivo situation. However, parameters such as smear layer formation and morphological conditions vary considerably, thus, parameter standardization is practically not given. Research methodology advantages such as time saving, standardized parameters and reproducibility of this in vitro model were the main reasons for its employment in this investigation. A further advantage of this research model was that Eppendorf tubes were chosen instead of human root canals or endodontic-blocks since they did not need to be mechanically prepared to a standardized size and sterilized. A further rationale for the employment of this research methodology is that the aim of the investigation was to compare the bactericidal potential of PAA against other irrigating solutions. Therefore, the need of closer in vivo situation was not considered a primary parameter.

Further research methodologies considerations are that the agar diffusion assay can be used to obtain preliminary antimicrobial potential evidence of an agent against a microbial strain. However, it is influenced by factors such as the diffusion rate of the agent and buffering capacity of the agar used in the test, thus, limiting comparisons between different agents or microorganisms. Another disadvantage is the time the microorganisms are exposed to the agent (at least 24 h), which does not resemble an endodontic clinical situation in which a root canal irrigant would be in contact with any residual bacteria. In contrast, in the direct contact test, a clinical exposure time can be reproduced, after which the agent can be removed and the number of viable microorganisms can be determined by various methods, including the determination of colony forming units to reflect the organisms which are still able to reproduce, thus, spreading and recolonize the root canal system. Nevertheless, when examining a root canal irrigation solution (PAA) that has not been previously in vivo investigated at such concentrations, thus, the agar diffusion test served as a starting point to compare its antibacterial efficacy with that of NaOCl and CHX. *E. faecalis* (DSM 20478) is commonly found in unsuccessful root canal treatments or in cases of persistent inflammation [13, 26], consequently, the effectiveness of root canal irrigation solutions on this bacterium has been investigated to a great extent [7, 9, 17, 27]. *P. micra* (DSM 20468) was chosen since it often appears in periapical infections and causes clinical symptoms [12, 22, 23, 26].

A similar bactericidal efficacy of PAA [8] and NaOCl [28] with the agar diffusion method, as well as NaOCl compared with CHX or EDTA [29–33] with similar concentrations with the direct method have been reported. The agar diffusion test results in this research showed that all root canal irrigation solutions in all concentrations formed inhibition zones with both bacteria investigated. PAA showed relatively large inhibition zones against *E. faecalis* and relatively smaller ones against *P. micra*. To the best of our knowledge, no agar diffusion investigations are available in which PAA had been used against *E. faecalis* or *P. micra*. The largest inhibition zone diameters against *E. faecalis* occurred decreasingly with 5.0, 3.0 and 1.0% NaOCl which are similar to other reports [28, 30, 32]. In the *P. micra* group, relatively smaller inhibition zones formed; yet, they did not increase with the PAA concentration. The same inhibitory effect of NaOCl against *E. faecalis* and *P. micra*; however, smaller inhibitions zones, as in other investigations [29, 31, 33], were observed. These differences could be explained with the research methodology differences (steel cylinders instead of paper discs and/or a shorter contact time). Alike other researches [29, 32], the inhibition zone diameters for CHX against *E. faecalis* also increased with the concentration incrementation. An inhibitory effect producing smaller inhibition zones was reported with the steel cylinder methodology [31, 33].

It is reasonable to assume a clinical contact time is intrinsically lower than the one of the agar diffusion tests of this research. Thus, the antimicrobial potential of PAA, compared with the one of NaOCl and CHX, was determined also in direct contact of the irrigating solutions with *E. faecalis* and *P. micra*. Various in vitro studies for PAA (0.5, 1 and 2%), NaOCI (0.5–5.25%) or CHX (0.2, 1, 2%) in different concentrations with different research protocols have been performed with direct contact against *E. faecalis* [27], on biofilms from root canal isolates [5] and with bacteria from biofilms [4, 8, 9, 33]. In this investigation, *E. faecalis* and *P. micra* bacterial pellets obtained from centrifuged bacterial suspensions were suspended in the irrigating solutions, simulating a clinical situation as close as possible, and left in contact with the irrigation solutions for 5 min. After centrifugation the viable bacterial counts were determined, thus, possible influencing factors inherent with the agar medium were eliminated. Furthermore, CHX can be inactivated by proteins present in the medium. The results obtained showed that no viable bacteria were observed after exposure to any of the root canal irrigation solutions used at their different concentrations. *E. faecalis* suspensions in direct contact with 1.0% PAA between 30 s and 10 min yielded similar results to the ones of this investigation [8]. Siqueira et al. [28] reported a similar antibacterial potential (97.1–99.9%) of NaOCl (at 1.0, 2.5, and 5.25% concentrations) with the same *E. faecalis* strain. This neglectable discrepancy might be due to bacteria accumulated in dentinal tubules, whereas in this research this possibility was not given. Further similar results [8, 9, 32] with CHX and NaOCl in similar concentrations with *E. faecalis* have been reported. Similar to the results of this investigation, 0.5 and 1.0% CHX eliminated *E. faecalis* after 5 min [32]; however, 0.12% CHX did not eliminate and 0.2 and 1.0% CHX (gel) eliminated *E. faecalis* only after 2 h and 1 min, respectively [27]. The inclusion of a commercial CHX-solution (0.12%) was implemented taking into consideration that it has become a common clinical practice in several European countries. These differences may be explained through the use of a different *E. faecalis* strain. The antimicrobial efficacy of NaOCl and CHX has been investigated also in biofilms including among others *E. faecalis* and *P. micra* [5]. As in this investigation, NaOCl eliminated both *E. faecalis* and *P. micra*, whereas CHX eliminated *P. micra*; yet, not *E. faecalis*. This could be due to the employment of isolates from infected root canals [26] and/or to CHX's inability to penetrate into deep layers of thick biofilms [6]. The antimicrobial efficacy of 2.0% PAA and CHX and 2.5% NaOCl on dentin blocks containing *E. faecalis* biofilms after a 3-min contact produced a 100% (NaOCl), 75% (PAA) and 66% (CHX) non-viable cell count [4, 34]. These tendencies are, to a certain extent, similar to the results obtained in this research and support the antimicrobial enhancement of NaOCl through its tissue dissolving potential as well as the reduced effect of CHX to penetrate into deeper regions.

A complete bacteria removal, among others, is of outmost importance to ensure a long-term endodontic treatment success. However, a complete bacteria removal can only be aimed for; yet, not completely achieved, by means of a mechanical and chemical preparation of the root canal system [2]. Furthermore, EDTA is commonly used, as an alternating irrigating solution, to enhance the smear layer removal, which is desirable in order to achieve a hermetic seal of the root canal system [35]. Yet, an inhibitory effect of EDTA on NaOCl has been in vitro observed [2]. It has been reported that EDTA and PAA can be used as root canal irrigating solutions since both possess smear layer dissolution properties [11, 36, 37]; however, the authors [11, 36] also report a root canal dentine decalcifying effect.

The results of this investigation showed that PAA was not able to reach the antimicrobial potential of NaOCl with the two bacteria investigated and it does not have the ability to dissolve tissue as NaOCl [10], thus, it would not be advisable to substitute the use of NaOCl with PAA. However, its routinely employment during endodontic treatment could be considered after elucidating if PAA could replace EDTA as a smear layer removal enhancement as its antibacterial properties would support this possibility. The results of this research support the null hypothesis that established that PAA would have a similar bactericidal potential against *E. faecalis* and *P. micra* as the one of CHX. Despite of, PAA could be recommended as a substitute of CHX and EDTA since the in vitro antibacterial properties of PAA and CHX are similar against *E. faecalis* and *P. micra*, PAA has a smear layer removal potential [38] and its bactericidal effect against *E. faecalis* is higher than the one of EDTA [9]. A further potential advantage of PAA, when compared with CHX is a clinical disadvantage when using CHX in combination with NaOCl. The possible production of a precipitate, thus, concomitant effects, demand surplus supplementary handling precautions during endodontic irrigation [39]. A clinically oriented investigation comparing the overall efficacy/advantages of PAA, CXH and EDTA should be the subject of further investigations.

## Conclusions

Peracetic acid (PAA; 0.5, 1.0 and 2.0%) showed a similar antibacterial efficacy as the one of NaOCl (1.0, 3.0, and 5.0%) and CHX (0.12, 0.2 and 2.0%) when in direct contact with *E. faecalis* and *P. micra*.

In the agar diffusion test, PAA showed a similar antibacterial efficacy as the one of CHX and a lower one when comparing 1.0% PAA against 3.0% NaOCl and 2.0% PAA against 5.0% NaOCl with *E. faecalis*.

## Data Availability

The datasets used and/or analyzed during the current study are available from the corresponding author on reasonable request.
