# Correction: Antibacterial efficacy of peracetic acid in comparison with sodium hypochlorite or chlorhexidine against *Enterococcus faecalis* and *Parvimonas micra*

**DOI:** 10.1186/s12903-022-02204-3

**Published:** 2022-05-10

**Authors:** Benjamín Briseño-Marroquín, Angelika Callaway, Natascha Gol Shalamzari, Thomas Gerhard Wolf

**Affiliations:** 1 Department of Restorative, Preventive and Pediatric Dentistry, School of Dental Medicine, University of Bern, Freiburgstrasse 7, 3010 Bern, Switzerland; 2 Department of Periodontology and Operative Dentistry, University Medical Center of the Johannes Gutenberg-University, Mainz, Germany

## Correction to: BMC Oral Health (2022) 22:119 10.1186/s12903-022-02148-8

In this article the wrong figures appeared due to a database error as figures 1 and 2; the updated Figs. 1 and 2 should have appeared as shown below.

**Fig. 1:**
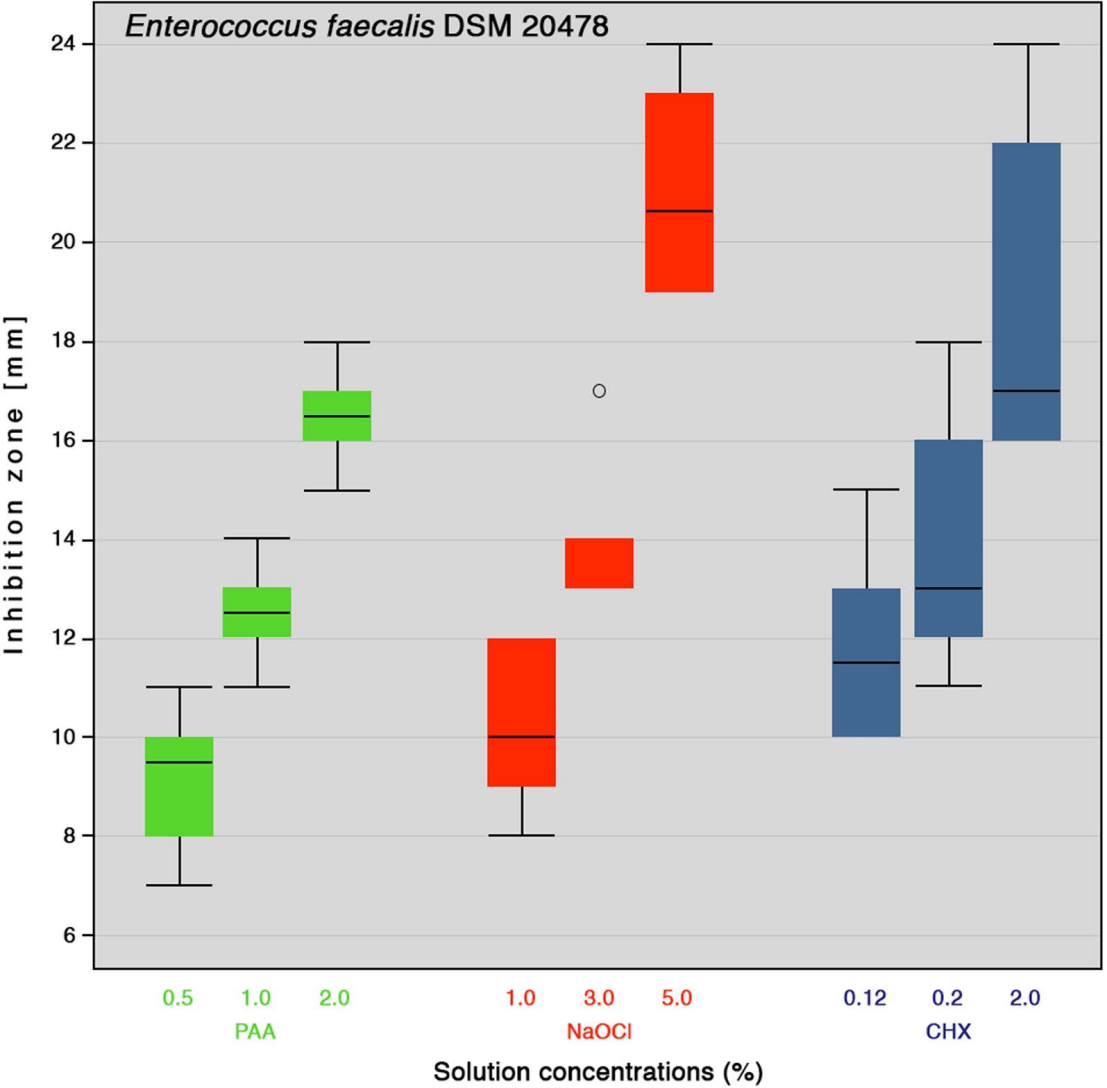
Inhibition zone diameters boxplot of different concentrations of peracetic acid (PAA), sodium hypochlorite (NaOCl) and chlorhexidine digluconate (CHX) against *E. faecalis* (n = 6). Medians are shown as lines inside the boxes, 25th and 75th percentiles as boxes, maximum and minimum values as whiskers, and outliers as circle on the plot

**Fig. 2:**
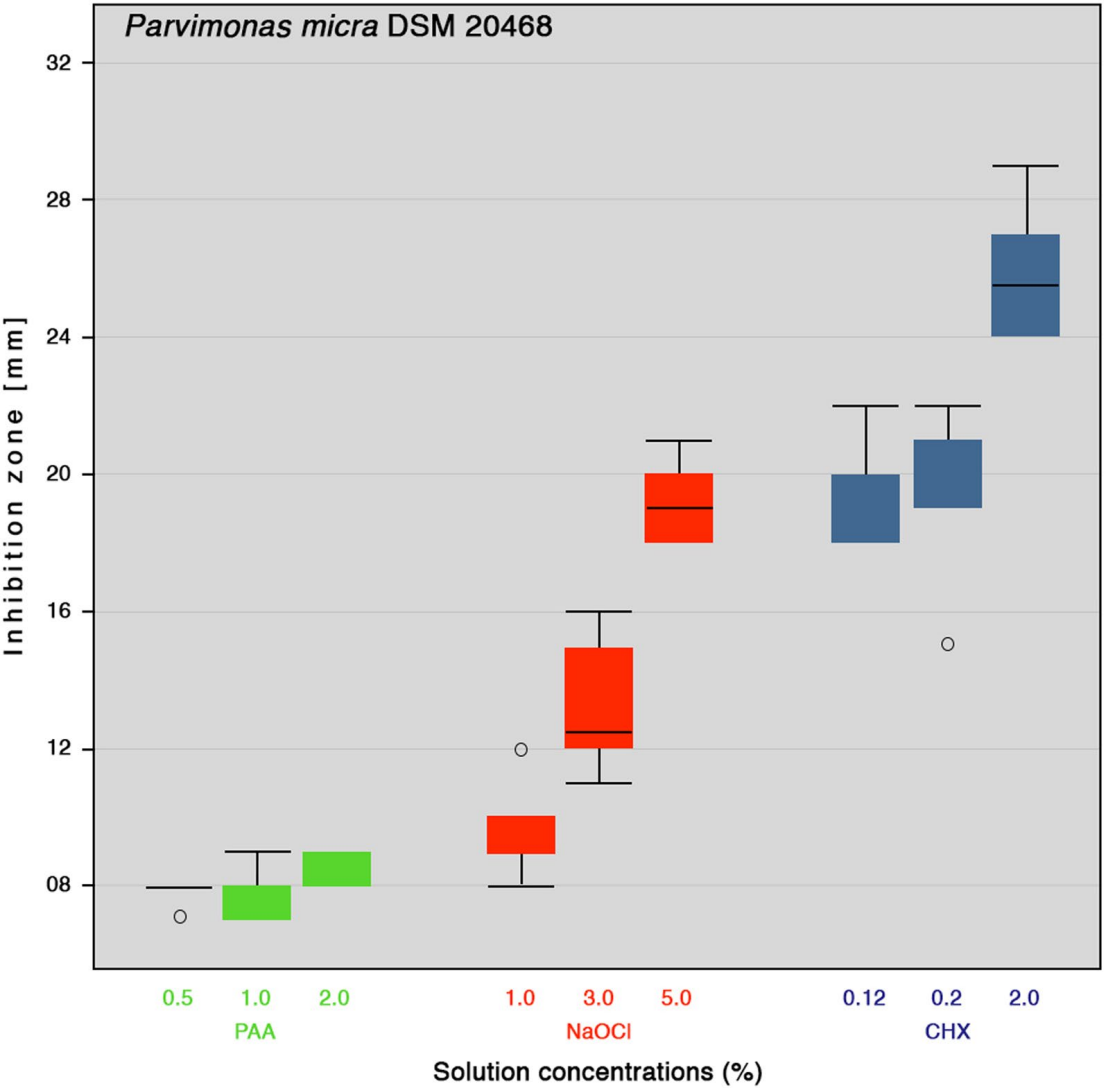
Inhibition zone diameters boxplot of different concentrations of peracetic acid (PAA), sodium hypochlorite (NaOCl) and chlorhexidine digluconate (CHX) against *P. micra* (n = 6). Medians are shown as lines inside the boxes, 25th and 75th percentiles as boxes, maximum and minimum values as whiskers, and outliers as circle on the plot

